# An infant with severe mitral valve regurgitation and aortic stenosis

**DOI:** 10.1016/j.xjtc.2026.102194

**Published:** 2026-01-09

**Authors:** Igor E. Konstantinov, Carolina Freire Rodrigues, Sergei I. Konstantinov, Amine Mazine

**Affiliations:** a Department of Cardiothoracic Surgery, Royal Children's Hospital, Melbourne, Australia; b Department of Paediatrics, University of Melbourne, Melbourne, Australia; c Heart Research Group, Murdoch Children's Research Institute, Melbourne, Australia; d Melbourne Centre for Cardiovascular Genomics and Regenerative Medicine, Melbourne, Australia

**Figure:**
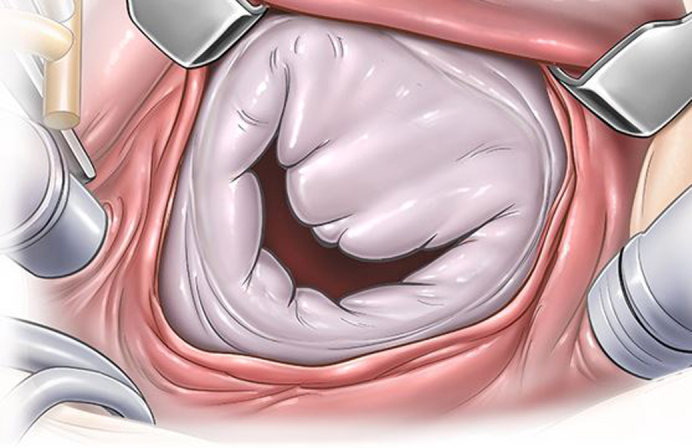
Severe mitral valve annulus dilatation in an infant.

**Central Message**

The technique described achieves reduction and stabilization of the mitral annulus in an infant.

Mitral valve repair in infants is challenging due to the frequent combination of type I and IIIb dysfunction, presenting with annular dilatation and leaflet restriction.^1^ The need for growth further limits the available repair techniques. Posterior annuloplasty with a band or suture can reduce the dimension of the annulus but interferes with growth.^1^

## Case Report

We describe the surgical management of a 5-month-old boy (weight 5.5 kg, height 60 cm, body surface area 0.29 m^2^) with severe mitral regurgitation and moderate aortic stenosis. The Royal Children's Hospital Human Research Ethics Committee (HREC/21/QCHQ/80891) approved the retrospective review on November 11, 2021, and the parents provided informed written consent for the publication of the data.

The echocardiogram demonstrated severe mitral regurgitation secondary to annular dilatation and restriction of the posterior leaflet. The jet was posteriorly directed. The aortic valve was trileaflet with thickened cusps and eccentric opening, resulting in moderate aortic stenosis, with a mean gradient of 30 mm Hg. The aortic valve annulus was normal and measured 8.2 mm by echocardiogram. There was normal biventricular function, but both the left atrium and left ventricle were severely dilated (Video 1).

**Figure 1:**
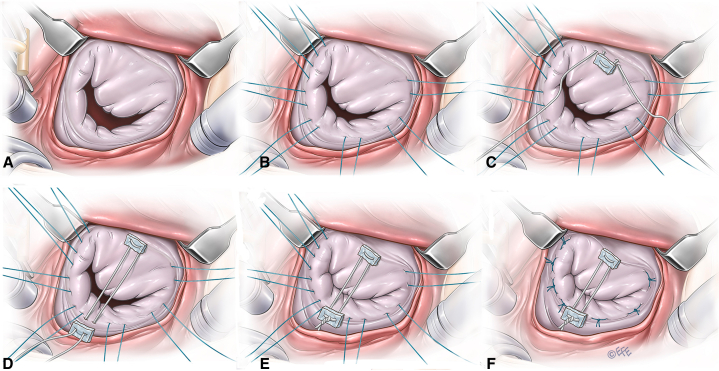
Mitral valve repair technique in an infant with severe annular dilatation. A, Severe mitral valve annular dilatation. B, Placement of interrupted annuloplasty sutures. C, Placement of the pledgetted polytetrafluoroethylene (Gore-Tex) suture at the anterior mitral annulus. D, Placement of the polytetrafluoroethylene (Gore-Tex) suture at the posterior annulus. E, Tying of the polytetrafluoroethylene (Gore-Tex) suture to reduce anteroposterior dimension of the annulus. F, Tying the annuloplasty sutures.

Intraoperatively, the aortic valve allowed passage of a size 8-mm Hegar dilator, whereas normal estimated size was 7.5 mm in diameter for this weight. The aortic valve showed near complete fusion of 2 commissures (Video 1). Therefore, commissurotomy was performed, and this allowed passage of a size 11-mm Hegar dilator. The mitral valve annulus was severely dilated, allowing passage of a 21-mm Hegar dilator, whereas normal estimated diameter for his weight was 12 mm. Seven 3/0 Dacron sutures were placed at the posterior annulus to decrease the annulus size. In addition, a pledgetted CV-3 polytetrafluoroethylene (Gore-Tex; WL Gore & Associates) suture was placed from the anterior annulus to the posterior annulus to decrease the anteroposterior dimension of the mitral annulus (Figure 1).

Aortic crossclamp time was 60 minutes, and cardiopulmonary bypass time was 79 minutes. The postoperative echocardiogram demonstrated mild mitral regurgitation and no aortic stenosis or aortic insufficiency. The patient is asymptomatic and thriving at 2 years of age; the echocardiogram findings are unchanged.

## Discussion

Aortic valve repair in neonates and infants has been well described and can be achieved even in the most dysplastic valves.^2^, ^3^, ^4^ Conversely, mitral valve repair in this age group is not well established.^5^ Furthermore, mitral valve replacement in children, and particularly in small infants, is associated with high mortality and reoperation rate.^6^ Reduction of the mitral valve annulus in small children is challenging^7^ because it must provide significant reduction while allowing for growth of the annulus. Therefore, placing an annuloplasty band would result in early reoperation as the patient grows, whereas plication of the annulus with interrupted sutures alone might not allow sufficient reduction and result in early recurrence of mitral regurgitation in a child with severe dilatation of the mitral annulus. Thus, in the infant described, we elected to place an additional polytetrafluoroethylene (Gore-Tex) suture to reduce the anteroposterior dimension. This approach provided an excellent immediate result. As the child grows, it will be relatively easy to remove the polytetrafluoroethylene (Gore-Tex) suture and perform a conventional annuloplasty. Because the polytetrafluoroethylene (Gore-Tex) suture is above the area of coaptation of the mitral valve leaflets and does not have contact with them, we expect that the growth of the mitral valve leaflets will remain unaffected. To the best of our knowledge, such a combination of mitral valve and aortic valve repair technique has never been described in a small infant with severe mitral valve annular and left heart chambers dilatation.

## Conclusions

The technique described resulted in an excellent early outcome in an infant with severe mitral insufficiency and aortic stenosis.

## Conflict of Interest Statement

The authors reported no conflicts of interest.

The *Journal* policy requires editors and reviewers to disclose conflicts of interest and to decline handling or reviewing manuscripts for which they may have a conflict of interest. The editors and reviewers of this article have no conflicts of interest.

## Supplementary Data

Video 1 The senior author explains perioperative findings and procedures. Video available at: https://www.jtcvs.org/article/S2666-2507(26)00001-5/fulltext.
