# Supplementary figures and images for: An infant with severe mitral valve regurgitation and aortic stenosis
Source: JTCVS Tech. 2026 Jan 9;36:102194. doi: 10.1016/j.xjtc.2026.102194 (PMC13069546; doi:10.1016/j.xjtc.2026.102194)

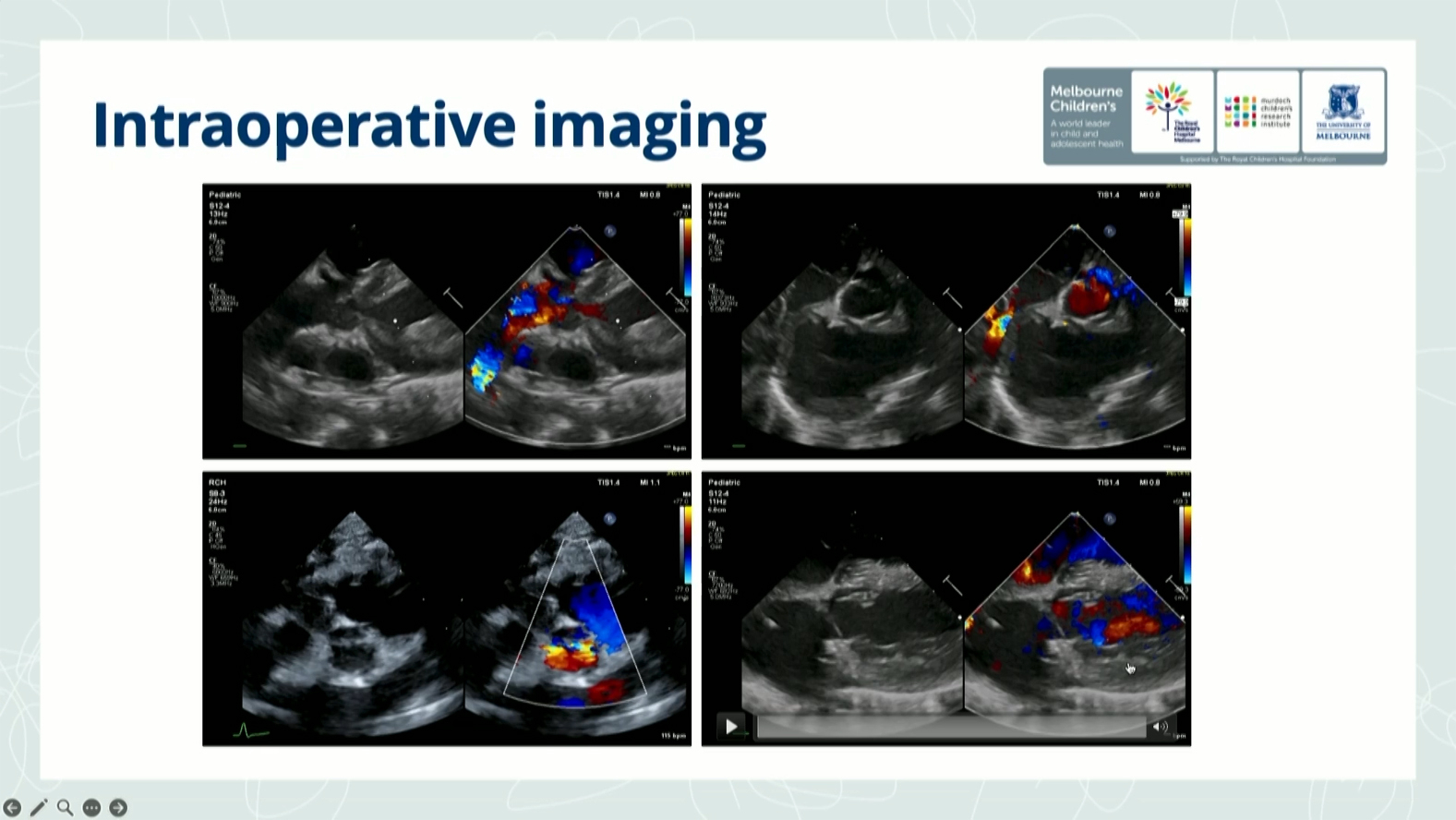

Supplement: Video 1 — The senior author explains perioperative findings and procedures. Video available at: https://www.jtcvs.org/article/S2666-2507(26)00001-5/fulltext. [file fx2.jpg]
